# A Rare Case of Traumatic Acute Pronator Syndrome in the Setting of Anticoagulation Therapy

**DOI:** 10.7759/cureus.36931

**Published:** 2023-03-30

**Authors:** Krystle R Tuano, Marlie H Fisher, Jerry H Yang, Mickey J Gordon

**Affiliations:** 1 Department of Surgery, University of Colorado Anschutz Medical Campus, Aurora, USA

**Keywords:** trauma, hematoma, anticoagulation, compression neuropathy, pronator syndrome

## Abstract

Pronator syndrome (PS) is a rare type of peripheral compression neuropathy in which the median nerve becomes entrapped as it passes through the pronator teres muscle at the proximal forearm. We report an unusual case of acute PS in a 78-year-old patient on warfarin who presented after traumatic forearm injury with forearm swelling, pain, and paresthesias. After emergent nerve decompression and hematoma evacuation, the patient regained near complete recovery of median nerve function six months after diagnosis and treatment.

## Introduction

Pronator syndrome (PS) is a compressive neuropathy of the median nerve in the region of the pronator teres [1]. The presentation is similar to that of carpal tunnel syndrome (CTS) making the diagnosis difficult and controversial [2,3]. Acute CTS is well-described in the literature [4], including multiple cases caused by hemorrhage into the carpal tunnel in patients on anticoagulation therapy, as opposed to acute PS, which is rarely reported [5-8]. Here we present a case of acute PS due to blunt traumatic injury in a patient on anticoagulation therapy requiring emergency surgical decompression.

## Case presentation

**Figure 1:**
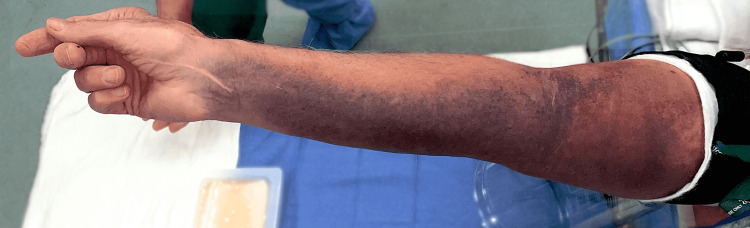
Preoperative photograph demonstrating weakness of AIN innervated muscles. AIN: anterior interosseous nerve

A 78-year-old right-hand-dominant male who was anticoagulated with warfarin after mitral valve repair and aortic root replacement presented to the emergency department (ED) following trauma to the right upper extremity while closing the tailgate of his truck five days prior to presentation. On initial evaluation, he was diagnosed with a proximal biceps tendon rupture and advised to withhold anticoagulation, when and if possible (international normalized ratio {INR}: 2.8), to elevate the extremity, and he was discharged home with strict return precautions. The patient subsequently developed worsening forearm pain, swelling, and paresthesias over the next 24 hours and returned to the ED. On examination, he had weakness in the anterior interosseous nerve (AIN) innervated muscles, with complete loss of flexion at the thumb interphalangeal joint, index finger proximal interphalangeal joint, and distal interphalangeal joints (Figure 1).

**Figure 2:**
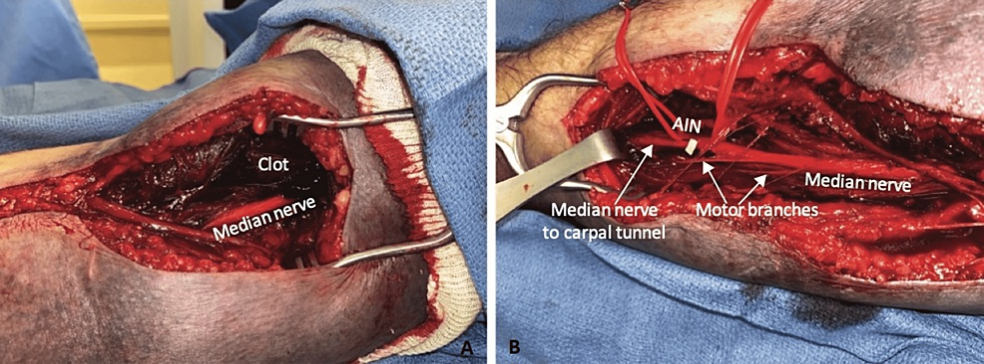
Intraoperative photographs of pronator tunnel release. The images show (A) visualization of hematoma and the median nerve and (B) illustration of nerve anatomy.

The patient was emergently taken to the operating room for decompression of the right median nerve (pronator tunnel release) and evacuation of hematoma after reversal of his anticoagulation with prothrombin complex concentrate and vitamin K. Approximately 100 mL of old clot was evacuated from the proximal forearm and the leading edge of the arch of the superficialis muscles was divided (Figures 2A, 2B). Anticoagulation was restarted on the evening of postoperative day one and occupational therapy was started on postoperative day two. Prior to discharge on postoperative day three, he had persistent numbness and tingling in the median nerve distribution, and difficulty with thumb and index finger flexion. Despite these deficits, the overall hand function has significantly improved since the initial presentation.

**Figure 3:**
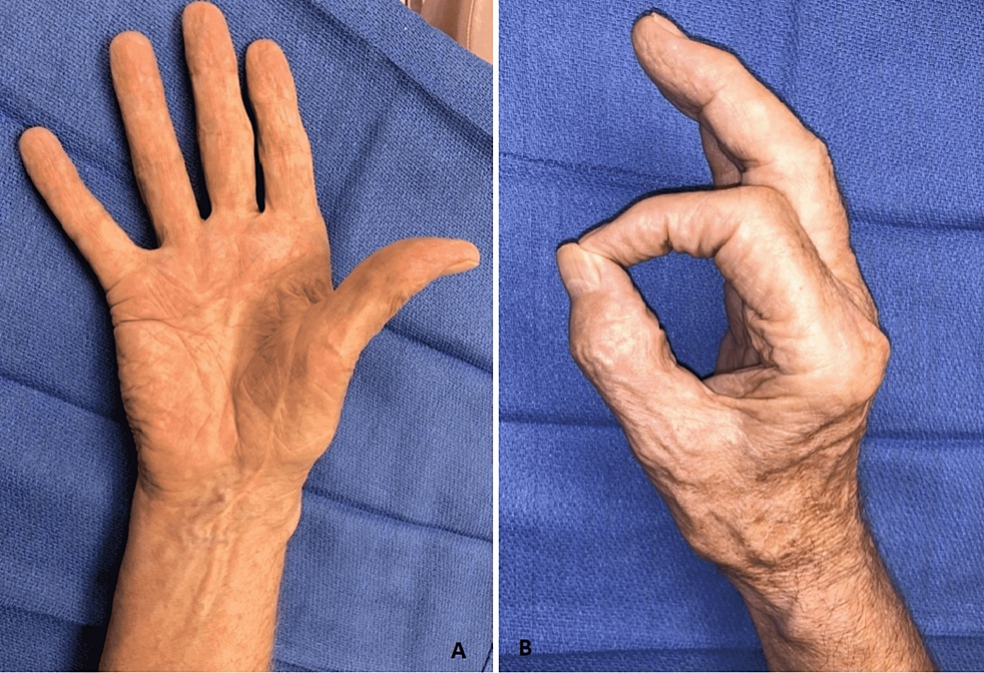
Postoperative photographs demonstrating recovery of AIN muscle function (A: palmar view and B: lateral view). AIN: anterior interosseous nerve

The patient continued to follow-up monthly with occupational therapy (OT), with functional goals met at approximately six months postoperatively (Figures 3A, 3B). At the time of his last postoperative visit, eight months after intervention, the patient had excellent recovery of function and was discharged from OT. The only residual deficits that the patient noted were minimal numbness at the tips of the index finger, long finger, and thumb, and difficulty with using a pump bottle for shampoo. Otherwise, the patient was able to perform his activities of daily living without significant issues. Recovery in flexion and extension angle of the thumb, index finger, and long finger were notable. For example, the index finger proximal interphalangeal joint active range of motion improved from 0-10 degrees to 0-95 degrees at three weeks vs. eight months postoperative visit.

## Discussion

Identifying compression of the median nerve in the proximal forearm can pose diagnostic and therapeutic challenges. Although rare, PS must remain on the differential diagnosis for numbness and paresthesia in the radial digits and volar forearm and wrist pain in the correct setting [9]. This constellation of symptoms can be distinguished from CTS, as PS nerve compression occurs proximal to the carpal tunnel. Absence of classical CTS findings, such as Tinel sign, exacerbation of symptoms with wrist flexion, and night symptoms suggest compression of the median nerve proximal to the carpal tunnel [9]. Another distinguishing feature of PS is paresthesia in the distribution of the palmar cutaneous branch of the median nerve. This region is spared in CTS as the palmar cutaneous branch courses superficially to innervate the palmar and thenar skin. The most common sign of PS is a positive pronator compression test, in which symptoms of pain and paresthesias are replicated by applying pressure proximal and lateral to the pronator teres muscle belly on the forearm [10]. As these conditions are difficult to distinguish clinically, therefore, critical diagnosis can often be delayed.

An alternative median nerve compression syndrome that should be considered with this symptomatology is anterior interosseous nerve (AIN) syndrome, an exceedingly rare cause of median nerve compression presenting with pure motor weakness [11]. Patients with AIN syndrome cannot make the “O” sign using the thumb and index finger on a physical examination. There have been reports of acute PS with features of AIN syndrome, as we describe in our patient [11]. Authors have advocated for acute proximal forearm median nerve compression to be viewed as a spectrum, on one end with classical sensory symptoms (PS), and on the other end with classical motor deficits (AIN) [11]. This viewpoint encourages surgeons to explore all potential sites of median nerve compression to avoid potential treatment failure.

PS is a rare and challenging diagnosis, and, consequently, studies comparing various surgical approaches are limited. Three anatomic spaces should be considered as possible points of compression - the supracondylar process of the humerus, heads of the pronator teres muscle, and the fibrous arch of the flexor superficialis [9,12]. The most common cause of PS is entrapment of the median nerve between the two heads of the pronator teres muscle, though other sites of nerve compression have been implicated in the literature [13,14]. Most surgeons opt for complete decompression of the median nerve and surgical exploration of all anatomic sites of potential compression, which has been shown to reduce or resolve symptoms for the majority of patients [11,15].

## Conclusions

Proximal release of the median nerve should be considered in the setting of trauma, particularly in patients on anticoagulation. It should also be considered in patients with clinical examination findings concerning paresthesias in the median nerve distribution and, in severe cases, with weakness in the AIN innervated muscles. Because symptoms can often overlap and the possibility of coexisting pathology, patients with acute CTS should be carefully evaluated for the possibility of PS needing surgical intervention.
